# The Air Sac Primordium of *Drosophila*: A Model for Invasive Development

**DOI:** 10.3390/ijms19072074

**Published:** 2018-07-17

**Authors:** Nathan Powers, Ajay Srivastava

**Affiliations:** Department of Biology and Biotechnology Center, Western Kentucky University, 1906 College Heights Boulevard, TCCW 351, Bowling Green, KY 42101, USA; nathaniel.powers848@topper.wku.edu

**Keywords:** air sac primordium, invasive development, *Drosophila*, tumor metastasis

## Abstract

The acquisition of invasive properties preceding tumor metastasis is critical for cancer progression. This phenomenon may result from mutagenic disruption of typical cell function, but recent evidence suggests that cancer cells frequently co-opt normal developmental programs to facilitate invasion as well. The signaling cascades that have been implicated present an obstacle to identifying effective therapeutic targets because of their complex nature and modulatory capacity through crosstalk with other pathways. Substantial efforts have been made to study invasive behavior during organogenesis in several organisms, but another model found in *Drosophila* *melanogaster* has not been thoroughly explored. The air sac primordium (ASP) appears to be a suitable candidate for investigating the genes and morphogens required for invasion due to the distinct overlap in the events that occur during its normal growth and the development of metastatic tumor cells. Among these events are the conversion of larval cells in the trachea into a population of mitotically active cells, reduced cell–cell contact along the leading edge of the ASP, and remodeling of the extracellular matrix (ECM) that surrounds the structure. Here, we summarize the development of ASPs and invasive behavior observed therein.

## 1. Introduction

Cancer has proven to be a notoriously difficult problem for researchers and clinicians to solve for a variety of reasons. Among these is tumor heterogeneity, which in part results from cancer cells’ capacity to shift between epithelial and mesenchymal states by hijacking normal developmental pathways [1,2,3]. For example, c-Jun N-terminal kinase (JNK) signaling and matrix metalloproteinases (MMPs) play demonstrable roles in both disc eversion, which is required for proper morphological development in *Drosophila*, and tumor invasion through control of basement membrane (BM) remodeling [3]. Border cells, as they migrate from the follicular epithelium of the *Drosophila* ovary toward the oocyte, similarly employ tactics akin to those adopted by tumor cells during metastasis. The two polar cells that lie at the center of this eight-cell cluster produce the activating ligand for Janus kinase/signal transducer and activator of transcription (JAK/STAT) signaling, unpaired (Upd), which subsequently initiates the collective migration of the surrounding border cells via JAK/STAT [4]. JAK/STAT signaling has been heavily implicated in tumor progression [5,6,7], as have other receptor tyrosine kinases (RTKs), such as those associated with platelet-derived growth factor (PGDF) [8], vascular endothelial growth factor (VEGF) [9,10], and epidermal growth factor (EGF) [11]. These latter RTKs are likewise critical for border cell migration by providing directional cues to long cellular extensions that generate the force required to navigate through nurse cells in front of the oocyte [12,13].

**Figure 1:**
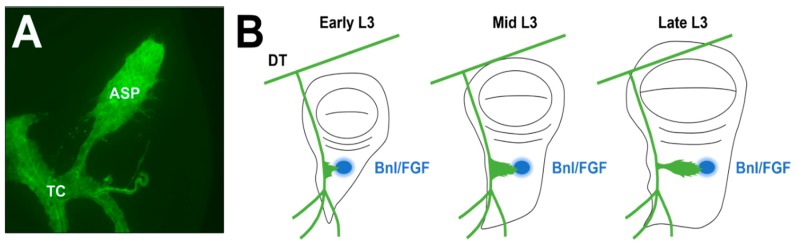
Wild-type ASP morphology and development. (**A**) and (**B**) The air sac primordium (ASP) develops from a population of mitotically active cells located along the transverse connective that divide and migrate toward the morphogen branchless/fibroblast growth factor (Bnl/FGF) throughout the third larval instar (L3) stage. Locations of the ASP, transverse connective (TC), dorsal trunk (DT), and Bnl/FGF (blue) are marked.

The threat of metastasis posed by the ability to acquire migratory properties is severe, as it is the leading cause of all cancer-related mortalities [14,15,16]. This fact makes preventing the mobilization of cancerous tissue a critically important subject for clinical research. Employing model organisms as an investigative tool has been, and continues to be, a useful approach for the discovery of novel treatment options [17]. While several models of invasive development in addition to those discussed above have been established previously [12,18,19,20,21], recent evidence suggests that the air sac primordium (ASP) of *Drosophila melanogaster* appears to be a worthwhile addition to this group and has notable unrealized potential. Compared to other, more complex models, *Drosophila* possesses some key advantages, including shorter generation times in addition to well-characterized genetic and developmental profiles. The ASP in particular serves as an interesting developmental model for several reasons. A short developmental period of only about five days is required for visualization of the ASP at its most advanced morphological stage [22,23], and established protocols exist for marking proteins, cells, and tissues effectively as a result of the aforementioned advantages inherent to using *Drosophila* as a model system. ASP are also known to exhibit a characteristic bud and stalk structure with a series of actin-rich filopodia protruding from said bud, which makes the distinguishing morphological features of this structure readily identifiable via confocal fluorescence microscopy (Figure 1A). Most importantly, ASP development involves three processes that are also required for tumor metastasis: re-initiation of a proliferative program in quiescent cells [24], downregulation of adherens junction proteins in tip cells [25], and extracellular matrix (ECM) remodeling [3,25,26,27]. Understanding the complex network of cellular signaling that orchestrates these events may yield new insights regarding metastasis and its prevention.

## 2. ASP Function and Development

Air sac primordia (ASPs) are the precursors to the dorsal thoracic air sacs found in adult *Drosophila*, which act to directly oxygenate the organism’s flight muscles via the interdigitation of tracheole bundles associated with the air sacs [22]. This role makes the ASP functionally analogous to that of the human lung, which similarly provides oxygen to all vascularized tissues. ASPs develop from a group of tracheoblasts in the transverse connective (TC) of the second tracheal metamere (Tr2) during the early third instar (L3) stage of larval development [23]. In contrast to the more derived method of development employed by many other holometabolous insects [28,29], *Drosophila* seems to utilize a more ancestral method of organogenesis with respect to ASPs [24]. Rather than eliminating larval cells through apoptosis and replacing them with a discrete population of imaginal cells, as proposed initially [30], the larval cells of Tr2 remain diploid and do not endoreplicate throughout larval development [24].

**Figure 2:**
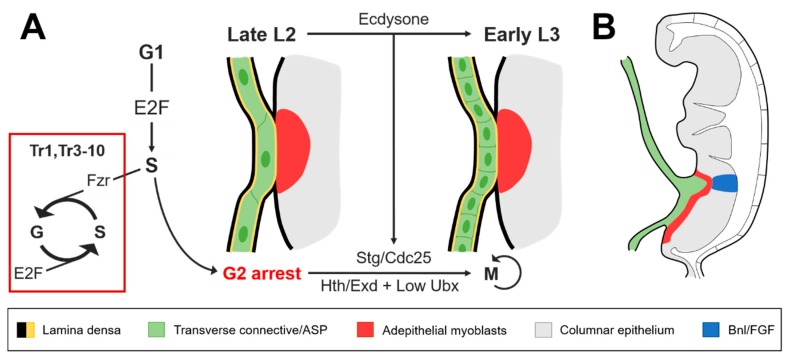
The transverse connective of the second tracheal metamere (Tr2) reenters the cell cycle at L3 and is invasively coupled with the wing imaginal disc prior to ASP development. (**A**) Permissive levels of homothorax (Hth)/extradenticle (Exd) and Ultrabithorax (Ubx) are required for ecdysone to activate string/Cdc25 (Stg/Cdc25) expression during the transition to L3. Stg/Cdc25 is necessary for larval cells in the trachea to reenter the cell cycle after the arrest at G2 that occurs during the second larval instar (L2) stage. In a high Hox background, fizzy-related (Fzr) and E2F cooperate to maintain polyploidy in the other tracheal metameres; (**B**) Lateral view of the wing imaginal disc during L3. Remodeling of the extracellular matrix (ECM) that separates the ASP from the Bnl/FGF expressed in the columnar epithelium is assisted through the invasive pairing of the transverse connective with the wing disc.

Recent evidence has pointed to the contributions of homeobox transcription factors in regulating entry and exit from these endocycles of continuous growth without division prior to metamorphosis [24,31]. Studying this relationship could be clinically beneficial considering that aberrant homeobox gene expression is associated with several cancers and that select candidates are predicted to serve as biomarkers for targeted therapy [32,33]. Furthermore, polyploid cells are reportedly less susceptible to traditional apoptotic pathways [34], which provides another mechanism whereby oncogenic tissues can circumvent cell-cycle checkpoints. This capacity to avoid cell death can become notably more pernicious if uncoupled from the arrested proliferative program associated with polyploidy. The cell-cycle regulators E2F and string/Cdc25 (Stg/Cdc25) are currently implicated as the driving forces behind the shifts from quiescence to G2 observed during the second larval instar (L2) and G2 to M phase in L3, respectively [31]. However, *stg* transcription is dependent upon the release of ecdysone that coincides with the transition between larval molts, and the process can be disrupted by the expression of fizzy-related (Fzr) [31], as is the case in the other tracheal metameres (Figure 2A). The absence of Fzr is necessary and sufficient for the reactivation of mitotic activity in the larval cells of Tr2 through Stg/Cdc25 [31], making its regulatory role paramount to initiating ASP growth via cell division [24]. Indeed, Fzr has previously been demonstrated to suppress tumor growth in mice [35] and is currently being investigated in human cell lines as a target for clinical application as well [36,37]. 

While endocycle exit helps initiate ASP development, morphogenesis is concomitantly directed by the fibroblast growth factor (FGF) homolog branchless (Bnl/FGF) through the action of filopodia that extend outward from distal tip cells and bind Bnl/FGF via the FGF receptor breathless (Btl/FGFR) [22,23,38] (Figure 1B). However, recent work indicates that the regulation of Ca^2+^ levels through the action of sarcoendoplasmic reticulum calcium transport ATPase (SERCA) at the tips of the ASP, trachea, and other tubules controls the timing of migration and branching prior to input from morphogens, including FGF [39]. The researchers found this regulation occurs within all germ layers and in vertebrate tissue as well, suggesting great potential for translating future mechanistic insights gained from studying the ASP and applying them in the search for novel remediation strategies. SERCA inhibitors have been proposed for the treatment of several cancer types [40,41], but cell-type specificity remains a challenge that might be overcome by developing and testing drug cocktails on ASPs demonstrating aberrant SERCA expression and searching for off-target effects in other tissues. Indeed, a similar screening approach was recently used to reveal synergy between trametinib and fluvastatin in both suppressing tumor formation and reducing whole-body toxicity associated with trametinib, using ASP and pupal air sac phenotypes to visually assay tumorigenicity [42].

The cells that secrete Bnl/FGF lie in the columnar epithelium of the wing imaginal disc while the population of tracheoblasts along the TC are embedded in the adepithelium among myoblasts destined to become the adult flight muscles [22]. Even after a calcium differential between “leading” (distal) and “lagging” (proximal) cells has been established to permit FGF signaling, this situation poses a challenge for the migration of ASPs toward the chemoattractant signal, as the cells must travel through the ECM surrounding the TC and adepithelium before arriving at the source of Bnl/FGF. However, the position of the TC with respect to the wing disc is rather conducive for invasive development, given that the thick layer of lamina densa encapsulating the trachea and wing imaginal disc thins out between the region where the ASP emerges along the TC and the adepithelial layer of the wing disc [26] (Figure 2B). The relationship between reduced ECM thickness and budding epithelia is also common to the ASP’s human analog, the lung, as well as other tissues that rely on ECM remodeling to facilitate branching morphogenesis, such as the intestine and mammary gland [43].

## 3. ECM Remodeling and ASP Invasion

**Figure 3:**
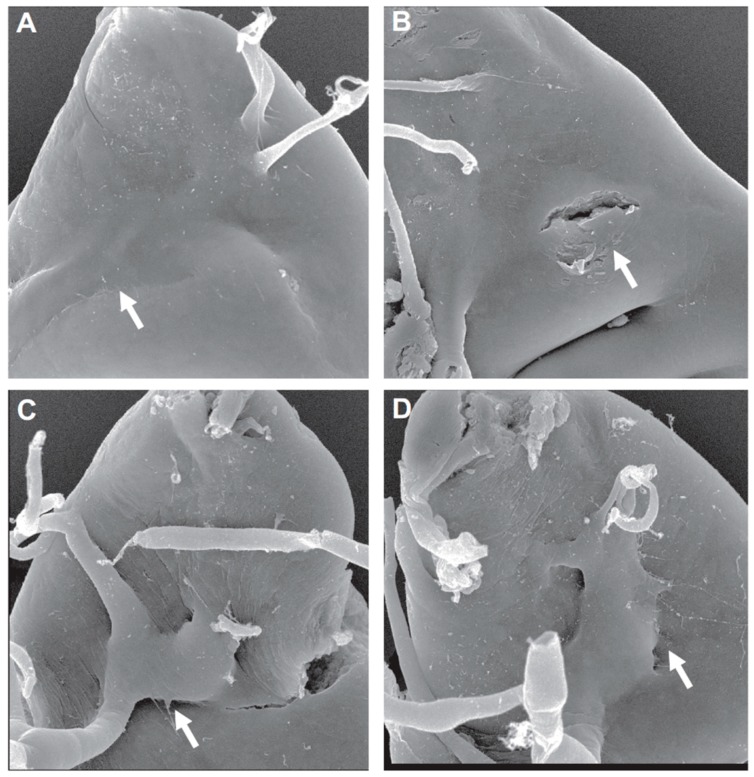
CP1/cathepsin-L (CTSL) knockdown suppresses ASP invasive behavior; (**A**–**D**) scanning electron micrographs (SEMs) of ASP locations (arrows) within the dorsal region of the wing imaginal disc. (**A**,**B**): The ASP is deeply embedded within the wing disc under wild-type conditions. (**C**,**D**): CP1/CTSL knockdown limits invasion, keeping the ASP in a more superficial position on the surface of the wing disc. Figure from [25], courtesy of the corresponding author.

Activated Btl/FGFR facilitates the expression of matrix metalloproteinase 2 (MMP2) in ASP tip cells [26,27]. MMP2 is an important protein for the degradation and remodeling of the ECM surrounding the developing ASP through collagen IV and perlecan turnover [3,26,27]. It has been hypothesized that MMP2-mediated ECM remodeling not only facilitates the invasive propagation of the ASP, but also releases some inhibitory signal that laterally prevents the expansion of tip cell fate by blocking Bnl/FGF binding sites in middle and stalk cells [27]. Another class of proteases, cathepsins, has been implicated in ECM remodeling around the ASP as well by evidence of hindered migratory capability in lines where CP1, an ortholog of cathepsin-L (CTSL), was knocked down (Figure 3) [25]. Interestingly, the ASP appears capable of outward growth in spite of its inability to penetrate the adepithelial layer of the wing disc, suggesting a role during invasion distinctly different from that of MMP2. Both of these proteolytic enzymes have been reported to serve as prognostic markers in several cancers, including breast [44,45], bladder [46,47], ovarian [48,49], prostate [50,51], lung [52,53], and pancreatic cancer [54,55]. However, the mechanisms by which CP1/CTSL facilitates ASP invasion and MMP2 prevents the lateral expansion of tip cell fate have yet to be investigated further. Regardless, the aforementioned thinning of ECM around the leading edge of the ASP plays another critical role unrelated to ECM remodeling. This thinner lamina densa makes the ECM highly permeable to FGF signaling, which allows for the initiation of a greater signaling response that regulates cell proliferation and survival [23,26,56], mirroring a similar mechanism required for human lung development [57,58,59,60].

## 4. Additional Regulators of ASP Development

**Figure 4:**
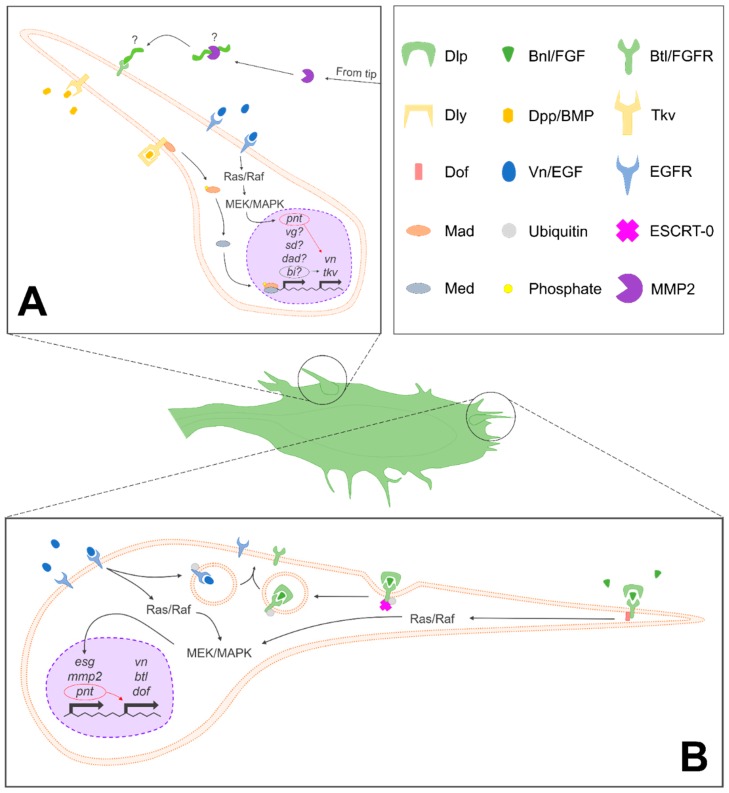
Signaling pathways involved in ASP development. (**A**) Summary of signals and pathways implicated in the development of lateral ASP cells, with speculative or unknown components denoted with “?”. (**B**) Summary of signals and pathways involved in the determination of tip cell fate and development. Endosomal sorting complex required for transport-0 (ESCRT-0)-mediated endocytic recycling is only depicted in (**B**), but this process occurs in (**A**) as well. A key with symbols used to denote various molecular actors is depicted in the top right panel, adjacent to (**A**). Dlp, dally-like; Bnl/FGF, branchless/fibroblast growth factor; Btl/FGFR, breathless/fibroblast growth factor receptor; Dly, dally; Dpp/BMP, decapentaplegic/bone morphogenetic protein; Tkv, thickveins; Dof, downstream of FGF; Vn/EGF, vein/epidermal growth factor; EGFR, epithelial growth factor receptor; Mad, mothers against dpp; ESCRT-0, endosomal sorting complex required for transport-0; Med, Medea; and MMP2, matrix metalloproteinase 2.

Several signaling pathways are known to play a role in the development of ASP in addition to FGF. EGF Receptor (EGFR) signaling is arguably the most important of this set for its role in the regulation of cell survival and proliferation [23,56,61]. It was recently found that FGF signaling induces vein (Vn/EGF) expression via the transcription factor pointedP2 (PntP2) [56]. This is notable because Vn initiates EGFR signaling in the ASP and induces a positive feedback loop that stimulates cell proliferation and survival in the stalk cells proximal to the tip cells [56]. Interestingly, the mechanism of action for this feedback loop is the activation of the mitogen-activated protein kinase (MAPK) signaling pathway by both Vn/EGF and Bnl/FGF; the key difference between stalk cells and the tip cells that induces their proliferation is that when MAPK is activated by Bnl/FGF, additional transcription factors, including pointedP1 (PntP1) and escargot (Esg), are expressed as well [22,23,56] (Figure 4). It has been proposed that Esg inhibits the proliferative genetic program activated by Vn/EGF-induced MAPK signaling and maintains the cell survival program, while PntP1 stimulates cell migration, together contributing to the determination of tip cell fate [56]. Downregulation of shotgun (Shg) and armadillo (Arm), the *Drosophila* homologs of E-cadherin and β-catenin, respectively, has also been observed in the tip cells of the ASP [25], but the contributing factors are currently unknown in spite of the role Esg plays in regulating Shg expression elsewhere in the tracheal system [62]. Recently, the *Drosophila* ortholog of dedicator of cytokinesis (DOCK) family proteins 3 and 4 (DOCK3/4), sponge (Spg), was reported to promote cell survival through MAPK signaling as well. The authors of the study suggest that this could be accomplished via the intermediate activation of p21-activated kinase (PAK), but this model currently lacks experimental evidence [63].

The localization and turnover of both Btl/FGFR and EGFR are regulated by Hrs and Stam, which together comprise the endosomal sorting complex required for transport-0 (ESCRT-0) complex [61] (Figure 4). While Hrs has been shown to downregulate EGFR signaling in embryonic development, it has been demonstrated that knockouts of Hrs and Stam result in inefficient FGF and EGFR signaling, as well as improperly localized Btl, collectively contributing to impaired ASP development [61]. This research highlighted the importance of endosomes in signal modulation at different points during development, continuing an observed trend in the regulation of RTK signaling [64]. Filopodia specific for the ortholog of bone morphogenetic proteins (BMPs) 2 and 4, decapentaplegic (Dpp/BMP), have been identified in the medial region of the ASP as well [38,65,66], but whether there are similar models for the regulation of this pathway and its contributions in the context of ASP development have yet to be determined (Figure 4). It has been proposed that two heparan sulfate proteoglycans (HSPGs) of the surrounding ECM, dally (Dly) and dally-like (Dlp), operate as co-activators of Dpp and FGF signaling, respectively [67,68] (Figure 4). However, another more recent model expands on this notion to suggest that the stratification of these ECM components also provides instructive cues and structural support for filopodia to facilitate guidance toward their target ligands [66]. This model parallels reported interactions between filopodia and ECM during neuronal development in avian and mammalian systems [69] but in cancer as well [70,71,72].

## 5. Conclusions and Future Directions

The dysregulation of proliferative programs has long been a widely recognized hallmark of cancer, and dedifferentiation has increasingly been implicated in the pathology of several cancers, including glioblastoma multiforme [73], colon cancer [74], and lung cancer [75]. Indeed, a recent mathematical model of mutation rates leading to carcinogenesis suggests that dedifferentiation of normal cells and loose homeostatic control are critical for hastening the onset of cancer [76]. In the interest of identifying key molecular contributors involved in these processes, attaining a greater understanding of how endocycle entry and exit in the *Drosophila* tracheal system are regulated via homeobox transcription factors could potentially highlight genes with functional human homologs for further investigation. Though significant differences exist between *Drosophila* and mammals regarding the frequency and nature of polyploidization [77], modeling the underlying mechanisms in fruit flies could contribute to our current understanding of polyploid giant cancer cells (PGCCs), as these have documented roles in breast [78] and colon cancers [79]. Elucidating the network of interactions required for Hox-mediated control of mitosis in this system could also apply more broadly to the progression of oncogenesis in other tissues, given the diverse roles that these transcription factors play [80].

Ras/Raf-MAPK [81], FGF [82], and EGFR signaling [11] all have well-documented roles in cancer progression as well, as is the case in ASP development. While the contributions of these signaling pathways are not unique to ASP development, the concurrence of their activities with ECM remodeling could enable the development of a holistic model for invasion that more accurately reflects both ASP migration and tumor metastasis, by extension, compared to other migrating cells. As such, drug screens targeting atypical signaling can reveal the impact of a treatment on processes ancillary to the one(s) targeted more readily when performed in this tissue than others. Of course, further exploration of how these signaling cascades function and cooperate with other pathways within the invasive context of ASP propagation should yield additional insights into what is required for their activity and regulation as well. For example, Notch signaling activity has also been reported to occur in the ASP, as evidenced by the expression of the pathway’s ligands, serrate (Ser) and delta [83,84]. It has been demonstrated that filopodia protruding from myoblasts in the adepithelium make contacts with both the wing imaginal disc and ASP in order to mediate wingless (Wg) signaling from the former and Notch signaling to the latter [84]. However, the mechanisms by which such signaling affect ASP development remain unknown. Even less is known about how Ser contributes to this structure’s morphogenesis, though Ser is known to be EGF-like and facilitate position-specific cell proliferation in the wing and haltere imaginal discs [85]. The transcription factors cut and knirps also have documented functions in morphogenesis—apoptosis inhibition [86] and Dpp signal mediation [87], respectively—but their role in ASP development is currently tentative and should be confirmed in order to derive a more complete model for invasive behavior.

The most imperative discoveries to be made regarding both tumor metastasis and ASP development may lie in the identification of novel regulators of ECM remodeling. In the ASP, many factors in this process require further investigation, especially the coupling of invasive migration with ECM degradation. Some contributors to ECM remodeling, including hypoxia [88], have yet to be investigated in the ASP at all. It has been demonstrated that hypoxic conditions promote autonomous sensitization to FGF in the tracheal system in order to vascularize oxygen-deprived tissues [89]. Assuming the ASP utilizes the same process, manipulating the ASP’s expression of the hypoxia-inducible factor (HIF)-α homolog and/or HIF-prolyl hydroxylase that regulate this response could potentially be used to imitate the hypoxic conditions found in malignant tumors. This would provide an additional set of drug targets that could be screened using the ASP as an invasive model.

The current literature on epithelial-mesenchymal transition (EMT) events and their role in metastasis is well established [90], but the determination of core molecular actors governing the loss of junctional contacts among tip cells in the ASP could shed light on ideal candidates for therapeutic intervention as well. Likewise, establishing how CP1/CTSL facilitates ASP invasion, characterizing the signal released by MMP2 to laterally inhibit tip cell fate, and determining how proteolytic enzymes cooperate during ASP development might provide new insights regarding ECM remodeling in human cancer progression. Resolving the issue of how Spg integrates with the other signals that regulate MAPK activity could also be rather useful, given that DOCK3 and DOCK4 were previously shown to be required for the migration of melanoma [91] and breast cancer [92] cells, respectively, via activation of the Rho GTPase Rac. Furthermore, Rac’s central role in actin remodeling [93] should make further study of Spg and the potential roles of other DOCK family proteins of interest for modeling invasive biomechanics in the ASP.

Developing a comprehensive framework for any biological process is a daunting task, and oncogenesis in humans is no exception. Nevertheless, with the power of the advanced molecular toolkit available for manipulating *Drosophila*, combined with its relative simplicity as a model organism, researchers can arrive at this endpoint far faster than we could ever imagine in humans. The convergence of many individually complex processes introduces countless redundancies and nuances. However, many of these confounding variables can be identified, simplified, and/or eliminated in *Drosophila* to obtain a clearer representation of the most fundamental elements and dynamics at play, which is ultimately what makes this goal worth pursuing.
